# Critical discussion of recently published claims of a causal relationship between bat decline and infant mortality

**DOI:** 10.1007/s00204-025-04142-9

**Published:** 2025-08-09

**Authors:** Philip Marx-Stoelting, Tewes Tralau, Veronika Städele, Stefanie Rotter, Vera Ritz, Jörg Rahnenführer, Jan G. Hengstler

**Affiliations:** 1 Department of Pesticides Safety, German Federal Institute for Risk Assessment (BfR), Berlin, Germany; 2 Department of Statistics, TU Dortmund University, Vogelpothsweg 87, 44227 Dortmund, Germany; 3 Department of Toxicology, Leibniz Research Centre for Working Environment and Human Factors (IfADo), Dortmund, Germany

It is undisputed that the use of insecticides must take place under conditions where adverse effects for humans are excluded. Therefore, the recently published study claiming a causal relationship between a 31.1% increase of insecticide use in USA to compensate for declines of bat populations in the affected counties and a 7.9% increase in human infant mortality seems alarming (Frank 2024). Such an increase—if true—would indeed be on a significant scale and would require extensive measures with considerable consequences for the efficacy and costs of food production. It is, therefore, important to carefully analyze such studies. Thus, we point-by-point evaluated all arguments made by Frank (2025) in response to our commentary (Marx-Stoelting et al. 2025). “First, the authors claim that causality cannot be inferred from observational data and present this misinformed opinion as fact. This ignores work that uses identical methods to study the effects of environmental change (Cheng et al. 2021; Currie et al. 2015; Dias et al. 2023; Emmett and Rubin 2025; Frank 2024; Marx-Stoelting et al. 2025; Popular information 2025). Even more so, their statement ignores the tremendous contribution that natural experiments have made in learning about a wide variety of outcomes, which culminated in awarding the 2021 Economics Nobel Prize to three scholars who developed those methods and “revolutionised empirical research in the social sciences and significantly improved the ability of the research community to answer questions of great importance to us all” (Raynor et al. 2021). Rejecting these type of analyses simply demonstrates the authors have not seriously engaged with understanding the methods and their assumptions.” (Frank 2025).

Response: It is not correct that we claimed in general that causality cannot be inferred from observational data (even though the concept of causality derived from observational data is controversially disputed in epistemology). Rather, we argued that the specific claim of a causal relationship between farmers in the USA increasing their insecticide use by 31.1% and an increase in human infant mortality of 7.9% (Frank 2024) is not justified based on the data presented and the methodology used. We argue that establishing causality requires a different approach and more investigation.

“The causal interpretation of the findings follows the research design that leverages a natural experiment, which approximates the ideal random manipulation of bat population levels and insecticide use. The research design relies on the fact that WNS emerged suddenly and unexpectedly, and from its first detection in 2006, it spread in a staggered fashion. This means that any confounding factor would also need to change in a systematic way that follows both the timing and the locations in which WNS emerges. The analysis in the SM demonstrates how such potential factors have not changed in this systematic manner (Tables S17, S18, S20, S21), and how they do not change the conclusions from the analysis when they are included as controls in the regressions (Tables S19, S22).” (Frank 2025).

Response: We do not agree with this argumentation for two reasons. The first is the inadequate control of confounders. The data reported in the supplement of Frank (2024) demonstrate that some of the analyzed confounding factors indeed changed following the emergence of the bat disease (white nose syndrome, WNS), in contrast to the statement made by Frank above. Figure S12 of the supplement (Frank 2024) shows that some maternal characteristics on average differed statistically significantly (CIs do not overlap zero) before and after WNS detection, e.g., rate of women aged 20–24 or rate of married women. Even though the magnitude of these differences in across-county means is rather small, these variables could, nevertheless, have influenced the analyses of infant mortality had they been included in the model as covariates as was done for other factors, such as crop composition and employment (Tables S19 and S22; Frank 2024). The supplement of the publication contains a large number of analyses, but the main analysis about infant survival does not (!) consider the maternal factors without giving any explanation for this omission. Therefore, the central conclusion was not adequately controlled for confounders and must be interpreted with caution. Moreover, previously published key factors influencing infant mortality, such as maternal income and minimum wage (Wolf et al. 2021), were not considered at all in the study of Frank (2024).

The second problem is the ecological fallacy, which remains even if the confounders described above would be sufficiently controlled at the county level. For an influential and relevant confounder, it is not necessary that the average value “needs to change in a systematic way that follows both the timing and the locations in which WNS emerges”. Importantly, confounding factors, such as maternal income or unemployment, act at the level of the individuals and should be adjusted for individuals and not for aggregated data (here at the average levels of counties). For illustration: imagine the income decreases in 50% of the mothers, so that they no longer can afford optimal healthcare causing increased infant mortality; this, by chance, occurs in a similar time period as a wildlife epidemic. For the second 50% of the mothers, income increases and healthcare (that was already good before) remains as before, leading to no change in infant mortality. In the total population, this leads to an average increase in infant mortality, but the average of the income remains unchanged, although income in reality represents an important confounder.

Moreover, we do not agree that the present study “leverages a natural experiment, which approximates the ideal random manipulation of bat population levels and insecticide use.” We do not see an “ideal random manipulation”, since the detection of WNS is an inadequate proxy for reduction in local bat populations, because it is unclear when exactly the bat population decreased and by which degree. Additionally, the author finds strong evidence for a non-random spatial spread of the disease. An analysis of bat populations was not performed in Frank 2024, so that the detection of WNS represents an imprecise estimate when the bat population really decreased.

“The authors demonstrate in their supporting documentation that they struggle with understanding the key structure of the regression specification and the comparisons made in the paper. The authors provide an illustration in Table 1 of their supplementary that fails to account for the fact that the regression I use in the analysis performs a double demeaning of the data. Each county has its mean outcome subtracted, and mean changes across time periods are absorbed in a variety of flexible ways. Those adjustments focus on changes within units, to test how outcomes change between the units of the two groups—exposed and unexposed to WNS—after units become treated (exposed to WNS) relative to how they were trending relative to untreated units (unexposed to WNS), with time periods where units are treated and untreated. In other words, aggregation is not a flaw in the statistical analysis. The authors of the comment fail to argue why this form of aggregation would invalidate the results. Especially in the context of the actual analysis performed in the study, and not a strawman comparison that does not resemble it.” (Frank 2025).

Response: Table 1 in Marx-Stoelting et al. (2025) is not meant to be an analog to the analyses conducted in Frank (2024), but simply an illustration of the "ecological fallacy" that shows why the use of aggregated data when making inferences about individual outcomes, such as infant mortality, is flawed (e.g., Robinson 1950; Wakefield 2008). We mention two citations here, but the “ecological fallacy”, and what can and cannot be gleamed from analysis of aggregated data, has been amply discussed in the literature. The relationship between two variables (A and B) can differ when means of groups are considered; however, A and B do not necessarily correlate at the individual level (for illustration, see Table 1 in Marx-Stoelting et al. 2025). The general argumentation applies irrespective of which statistical methodology is used. The “units” of analysis in Frank (2024) are US counties not individual mothers and their infants. Infant mortality is an outcome that is strongly influenced at the individual level, for example, economic factors and access to health care of the individual mothers. It is not justified to consider these factors of influence using county-aggregate measures.

It should also be mentioned that the “changes within units” before and after WNS detection (“exposure”) do not amount to statistical significance, since the 95% confidence intervals overlap zero for most of the individual years analyzed (see Fig. 2 of Frank 2024) and statistical significance is only achieved after application of a controversially discussed binning procedure (see below).

“The incorrect claim that the study should have estimated the direct relationship between infant mortality and insecticide use is deficient because it ignores the complexities of modeling how multiple different chemicals affect (likely non-linearly) human health. Because so many different insecticides might be responding to the reduction in biological pest control, the original analysis is ill-suited to estimate a dose–response function.” (Frank 2025).

Response: If a 31.1% increase of insecticide use caused (!) a 7.9% increase of infant mortality, as claimed by Frank (2024), a correlation between (increased) insecticide use and (increased) mortality over the years should be expected. Essentially, the author argues here that the association between a rough proxy for bat population decline and infant mortality, which are claimed to be causally connected by insecticide use, is better evidence for an effect of insecticide use on infant mortality than the direct investigation of the effect of insecticide use on infant mortality, because the methodology and measure of insecticide use are not suitable for modeling the relationship. If the currently used method of analysis does not lend itself to investigating this relationship, why not use different methodology? If human health is affected by multiple chemicals in complex ways, too complex to accurately model the direct relationship between insecticide use and infant mortality with the presented methodology and data, can the claim be made that the presented association of WNS emergence and insecticide use tells us anything about effects of insecticide use on human health?

It should also be considered that very high concentrations of insecticides should be detectable in the mothers and the blood and tissues of the deceased babies, if insecticides really were responsible for their deaths. It is not a convincing argument that many different insecticides exist and that dose–effect curves may be non-linear. Biomonitoring of insecticides in human body fluids and tissues and the assessment of internal concentrations represent well-established procedures in toxicology. It is not convincing to make severe claims such as the death of infants from insecticides, but to omit routine analyses for verification.

“The authors of the comment continue to err by ignoring that bat population declines are well proxied by WNS emergence because of the high lethality of the disease (Springborn et al. 2022);” (Frank 2025).

Response: It is unclear when and to which degree bat populations actually decline when WNS has been confirmed. The author estimates a median delay of 2 years (0–4; Fig. S6), but the spread in delays around this median will result in larger or smaller differences for many counties. This may be problematic, since the standard difference in difference analysis (DiD) as applied by Frank (2024) requires the precise timing of “treatment”, such as the passing of a law, etc. The unclear beginning of the “treatment” may critically influence the results as described in Marx-Stoelting et al. (2025).

“ignore the use of the time from initial exposure in each county to further proxy for more severe declines (Fig. 2);” (Frank 2025).

Response: It is unclear what the author means with this statement.

“fail to correctly interpret that maternal characteristics have no meaningful differences in WNS counties relative to non-WNS counties following the emergence of WNS (Fig. S12);” (Frank 2025).

Response: This has already been discussed above. Briefly, some maternal characteristics (confounders) differed significantly before and after WNS detection, and were not controlled for in the analysis for the main conclusion concerning infant mortality. Moreover, the above-described problem of the ecological fallacy may compromise the conclusion.

“incorrectly conflate following best practice approaches to the binning of the regression coefficients as “questionable”;” (Frank 2025).

Response: It indeed represents a complex problem that the author pooled (“binned”) data of time periods to reach statistical significance, while data of the individual years did not amount to statistical significance (Fig. 2; Frank 2024). To justify this “binning”, the author cites two references in the supplement of his study, references 92 and 64. Probably, Frank mixed up references 92 (Mnif et al. 2011) and 91 (Miller 2023) and means reference 91 (Frank 2024; Supplement, page 11). In the supplement, Frank writes that they are “following the suggestions of references 92 and 64” (Frank 2024; Supplement, page 11) and in his letter writes that he used “best practice approaches” (Frank 2025). However, these recommendations of “best practice approaches” cannot be found in the articles of Miller 2023 and Borusyak et al. 2024, but these authors rather critically discuss the risk of bias due to the binning or pooling of data. For example, Borusjak (2024) writes in his chapter “upward bias with binning” that “…the short-run biased weighting scheme due to binning explains nearly all the differences..” (p. 3276–3278; Borusjak, et al. 2024); also, Miller (2023) critically discusses the pooling of data: “The main risk is that the pooling might obscure features of the empirical results.” (p. 216; Miller 2023). This problem may indeed be relevant for the data of Frank (2024) presented in Fig. 2, because they show a trend for increasing insecticide use and increasing infant mortality from year 0 to 4, but the opposite constellation (increasing insecticide use but decreasing infant mortality) from year 4 to 6. Thus, the applied binning or pooling approach may be problematic and should not be named “best practice approach”.

“and outrageously claim that following a data use agreement that prevents me from sharing raw data on infant mortality accounts as “lack of data documentation.”” (Frank 2025).

Response: In fact, we wrote in our commentary that not providing the data “was not necessarily under the author’s control.” Nevertheless, the lack of availability of the data makes it impossible to replicate the findings. It remains difficult to understand why county-wise anonymized data on infant mortality should be so sensitive that publication must be prohibited.

In conclusion, considering the data and arguments provided, we are not convinced that the claim of causality between a compensatory increase of insecticide use and increased infant mortality is justified and we do not find the answer of Frank to our critiques convincing either.
